# Role of glucocorticoid receptor expression in Chronic Chagas Cardiomyopathy: implications for inflammation and cardiac hypertrophy

**DOI:** 10.3389/fendo.2025.1486772

**Published:** 2025-01-30

**Authors:** Florencia B. González, Antonella Pacini, Araceli Castro, Susana Lioi, Silvina R. Villar, Luciano D’Attilio, Rodolfo D. Leiva, Liliana Favaloro, Oscar A. Bottasso, Carlos A. Vigliano, Ana Rosa Pérez

**Affiliations:** ^1^ Instituto de Inmunología Clínica y Experimental de Rosario (IDICER-CONICET), Rosario, Argentina; ^2^ Facultad de Ciencias Médicas, Universidad Nacional de Rosario, Rosario, Argentina; ^3^ Instituto de Medicina Traslacional, Trasplante y Bioingeniería (IMeTTyB), Universidad Favaloro-CONICET, Buenos Aires, Argentina; ^4^ Laboratorio Central del Hospital Provincial del Centenario, Facultad de Ciencias Bioquímicas y Farmacéuticas, Universidad Nacional de Rosario, Rosario, Argentina; ^5^ Servicio de Cardiología, Sección Chagas del Hospital Provincial del Centenario, Rosario, Argentina; ^6^ Servicio de Insuficiencia Cardíaca y Trasplante Cardíaco, Hospital Universitario Fundación Favaloro, Buenos Aires, Argentina; ^7^ Servicio de Anatomía Patológica, Hospital Universitario de la Fundación Favaloro, Buenos Aires, Argentina

**Keywords:** glucocorticoid receptor, 11β-hydroxysteroid dehydrogenase type 1, cardiomyocyte, hypertrophy, chronic chagasic cardiomyopathy, ischemic cardiomyopathy

## Abstract

**Introduction:**

Chronic Chagasic Cardiomyopathy (CCC) has an infectious and inflammatory nature. Recent data also suggest an association with altered regulation of glucocorticoid (GC)-mediated circuits failing to control systemic inflammation. However, the involvement of glucocorticoid receptors (GR) and their isoforms have been unexplored.

**Materials and methods:**

The expression of GR-α/β isoforms, 11β-hydroxysteroid dehydrogenase type-1 (11β-HSD1), inflammatory cytokines, and the GC-regulated gene tristetraprolin (TTP) in peripheral blood mononuclear cells (PBMCs) as well as GR immunoreactivity in the myocardium from CCC individuals were evaluated by qPCR and immunohistochemistry respectively. Heart control samples with no evidence of structural heart disease and from ischemic cardiomyopathy patients were included. The presence of inflammatory infiltrates and fibrosis were also recorded.

**Results:**

GR-α was expressed similarly in the PBMCs from Co and CCC individuals, but 11β-HSD1 expression was increased only in CCC, conjointly with enhanced ratios of IL-6/TTP and IFN-γ/TTP. In the inflamed myocardium from CCC patients, positive GR expression correlated with the intensity of the inflammatory infiltrate and cardiac hypertrophy.

**Conclusion:**

The infectious and inflammatory nature of CCC pathology seems strongly connected with the expression of GR in cardiac tissue samples, providing a stimulating background for further studies addressed to elucidate the influence of GR expression and function on CCC pathophysiology and cardiomyocyte hypertrophy.

## Introduction

Chagas disease is caused by the parasite *T. cruzi*. Nearly 30% of infected individuals evolve to chronic and irreversible conditions, being chronic chagasic cardiomyopathy (CCC) the commonest clinical manifestation (1).

The pathophysiology underlying the establishment of CCC remains incompletely elucidated (2). The immune response is involved in the disease onset together with other potential factors such as metabolic and endocrine disorders, but their proper relevance remains uncertain. CCC seems to arise from two primary pathogenetic processes (1): myocardial damage directly caused by inflammation associated with parasitized cardiac fibers, and (2) myocardial injury resulting from an adverse or dysregulated immune response. It is believed that both mechanisms contribute to the progressive cardiac dysfunction observed in CCC. Regardless of the underlying cause, anti-inflammatory pathways activated by endogenous glucocorticoids (GC) through glucocorticoid receptor (GR) signaling can potentially modulate these processes, influencing the extent of myocardial damage and inflammation.

In this sense, earlier studies in patients with CCC showed increased levels of pro-inflammatory mediators in parallel with a dysregulated hypothalamic-pituitary-adrenal (HPA) axis activation, as revealed by low levels of cortisol and dehydroepiandrosterone sulfate (DHEA-S) (3). These findings suggest that the persistence of pro-inflammatory factors and the adverse endocrine anti-inflammatory *milieu* may play a role in the underlying mechanisms contributing to myocardial damage in CCC individuals.

Endogenous GCs regulate physiological processes such as development, metabolism, immunity, and cardiovascular function (4). Concerning the immune system, enhanced levels of GCs exert anti-inflammatory and immunosuppressive effects. GCs can inhibit leukocyte traffic and thereby the access of leukocytes to the inflamed sites. Furthermore, they can interfere with immune cell function suppressing cytokine production and other factors involved in inflammation. GCs also inhibit the Th17 pattern and turn the Th1/Th2 balance favoring an anti-inflammatory environment (5). Additionally, GCs affect the expression of tristetraprolin (TTP), an RNA-binding protein known for binding to AU-rich elements in the 3’-untranslated region of pro-inflammatory transcripts that accelerates their degradation, indicating a key role in controlling inflammation (6). Indeed, TTP-deficient mice present an excessive accumulation of pro-inflammatory cytokine mRNAs and their encoded proteins, leading to severe systemic inflammation (7, 8).

The anti-inflammatory effects of GCs are mainly mediated by the GR. This receptor belongs to the nuclear receptor superfamily of transcription factors and is constitutively and ubiquitously expressed. GR-α and GR-β isoforms result from an alternative splicing process, differing only in their C-terminal-end (9, 10). GR-α acts as the primary receptor accountable for GCs actions, while GR-β is associated with the disruption of GRα-mediated functions. GR-β is constitutively found in the nucleus, it cannot bind to GCs and was found to be up-regulated in inflammatory contexts, playing a role in GCs resistance in several diseases (4). In the absence of intracellular bioactive GCs, the GR finds itself as a monomer in the cytoplasm.

Cytoplasmic GC bioavailability is regulated by the balance of their active (cortisol) and inactive (cortisone) forms. Two enzymes are responsible for the transformation between these forms: 11β-hydroxysteroid dehydrogenase type-1 (11β-HSD1) mainly catalyzes the transformation of cortisone into cortisol, while 11β-HSD2 performs the reverse process. Hence, the equilibrium between both forms of GCs also regulates their activity (11, 12). After GC-binding, the GR suffers a conformational change exposing nuclear localization signals that carry the GR through the nuclear pore into the nucleus, where transcriptionally activate or repress gene expression (11).

Regarding cardiac homeostasis, GCs are involved in the maintenance of myocardial function, displaying an anti-apoptotic activity in contrast to the pro-apoptotic role over lymphocytes (13). This anti-apoptotic role depends on the regulation of GR downstream target genes and runs in parallel with cardiomyocyte hypertrophy (14, 15). Most evidence comes from transgenic models, where GR overexpression in cardiomyocytes induced electrocardiogram (ECG) abnormalities but not hypertrophy or fibrosis (16). Alternatively, GR signaling abrogation in the heart produces cardiac hypertrophy without fibrosis, deteriorated ventricular function, and leads to premature death and heart failure (17).

We hypothesize that the combination of low-normal cortisol levels and increased pro-inflammatory markers in *T. cruzi*-infected patients could harm heart function and promote inflammatory-driven CCC progression alongside disrupted GC signaling. Here, we investigated the possible participation of GR in the crosstalk between endocrine and immune systems in CCC patients and analyzed whether some surrogate of GC sensitivity, i.e., GR expression, may be modified in CCC subjects known to present myocardial inflammation and hypertrophy.

## Materials and methods

### Study population

A total of 51 participants were enrolled in the Cardiology Service at Hospital Centenario, Rosario, Argentina for the study procedures to be assessed in PBMCs. The cohort included 22 individuals with CCC, 14 individuals with ischemic cardiomyopathy (ICM) serving as controls for non-Chagasic cardiomyopathy, and 15 healthy volunteers matched for sex and age as additional controls (Co). Chagas disease diagnosis was based on at least 2 positive serological findings. Co and ICM subjects were seronegative to *T. cruzi*-specific tests. None of the participants were undergoing benznidazole/nifurtimox treatment or had additional pathological conditions. Exclusion criteria included other heart conditions, neuroendocrine, metabolic, or immunological disorders, as well as treatments involving hormones or immunomodulators. CCC (and comparably ICM) patients were included in the I-III categories of Kuschnir classification based on clinical, electrocardiographic, and echocardiographic findings (18). The clinical and demographic characteristics of patients and healthy subjects are shown in **Supplementary Table S1**.

Heart tissue samples from cardiac transplant patients with CCC (n=12) and ICM (n=6) were also collected at Fundación Favaloro Hospital, Argentina. Control heart samples (n=6), with no evidence of structural heart disease, were obtained from individuals who died from traumatic brain injury. ICM and CCC transplanted patients are included in functional classification III and IV of the New York Heart Association criteria (19). The clinical and demographic characteristics of these subjects are detailed in **Supplementary Table S2**.

### Plasma collection and hormone assays

Fasting blood samples were collected in EDTA tubes (8:00-10:00 a.m.), centrifuged at 2000 rpm for 30 min, and then, plasma was preserved at –70°C. Cortisol and DHEA-S were measured in duplicate by electrochemiluminescence (Roche Diagnostics).

### Mononuclear cell isolation

Peripheral blood mononuclear cells (PBMCs) were obtained from EDTA-treated blood. Cell suspensions were layered over a Ficoll-Paque-PLUS (GE-Healthcare) gradient and centrifuged at 400 g for 30 min at 25°C. The buffy-coat was washed twice, and 8.10^6^ cells were collected in TRI-Reagent^®^(MRC) for RNA isolation and stored at -80°C until needed.

### RNA isolation, cDNA synthesis, and qPCR

Total RNA from 5.10^6^ human PBMCs was isolated using TRI-Reagent^®^(MCR), according to the manufacturer’s recommendations. The cDNA was synthesized from 2ng of total RNA using RevertAid Reverse Transcriptase (Thermo-Fisher Scientific) and specie-specific primers for GR-α and GR-β, interleukin (IL)-1β, IL-6, interferon-γ (IFN-γ), tumor necrosis factor-α (TNF-α), 11β-HSD1 and TTP (**Supplementary Table S3**). PCR reactions were performed by using the Mix-5x-HOT-FIREPol^®^EvaGreen^®^ qPCR-Mix-Plus with ROX (Solis-BioDyne). mRNA level expression was determined by RT-qPCR, performed in a StepOnePlus PCR System (Applied Biosystems, USA). Data was normalized using human cyclophilin-A cDNA quantification.

### Histology and immunohistochemistry of cardiac tissue

Explanted hearts were weighed and fixed for 72h in 10% phosphate-buffered formaldehyde. Transmural sections of the whole circumference of both ventricles at a plane equidistant from the base to the apex were collected and embedded in paraffin. A 5 µm thick section from each region was stained with hematoxylin/eosin or Masson’s trichrome. Myocarditis diagnosis was defined according to the Dallas criteria (20). The maximal cardiomyocyte diameter was determined by transverse sections at the nuclear level (21). Cardiomyocyte nuclear size was measured on longitudinally oriented nuclei equidistant from the cell boundaries (22). The interventricular septum was selected for the analysis of inflammatory infiltrate numbers (23, 24), fibrosis (25, 26) and GR expression.

To analyze GR expression, staining with anti-GR (clone 41-BD#611226), was performed using an automated immune stainer (Benchmark Ultra, Ventana Medical Systems/Roche, USA). Briefly, formalin-fixed, paraffin-embedded (FFPE) tissue sections were cut in widths of 3 μM. After deparaffinization, slides were treated with cell conditioning reagent 1 (CC1, Roche Nr.950-124) for antigen retrieval. The OptiView IHC DAB Detection Kit (Roche Nr.760-700) was used for visualization following the manufacturer’s recommendations. Finally, slides were washed in distilled water, counterstained with hematoxylin and bluing reagent, dehydrated in descending order of alcohols, cleared in xylene, and cover-slipped Canada balsam (**Supplementary Table S4**). Image acquisitions (400X) for all measurements were performed using a Nikon-ECL microscope equipped with a Nikon Y-TV55 camera. GR expression in cardiomyocytes, quantification of inflammatory cells, and morphometric measurements of diameter to estimate hypertrophy and nuclear area were analyzed in 10 random fields (27). Fiji software was used for quantifications (28).

### Statistical analysis

Categorical variables are presented as percentages and were compared using chi-square or Fisher tests. Continuous variables are presented as means with standard deviations (SD) or medians with interquartile ranges (IQRs). The Kolmogorov-Smirnov test was used to examine variable distribution. Normally distributed variables were analyzed using one-way ANOVA with *post-hoc* Scheffé comparisons, while non-Gaussian distributed variables were analyzed using the Kruskal-Wallis test. Correlation analysis between demographic, clinical, and morphometric variables was performed using the Spearman correlation test. A *p*-value <0.05 was considered statistically significant. Statistical analyses were performed using SPSS 15.0 (SPSS Inc., USA) and GraphPad Prism 8.

### Ethics statement

Studies were conducted under the Helsinki Declaration and approved by the Institutional Ethics Committees of FCM-UNR (Res. N°2001/2012, 4977/2013), Hospital Centenario (Res. N° 282), and Fundación Favaloro (DDI (1331) 0616 CBE 605/16). All participants provided informed consent before inclusion.

## Results

### Myocardial involvement courses with a dysregulated HPA axis activation

**Figure 1:**
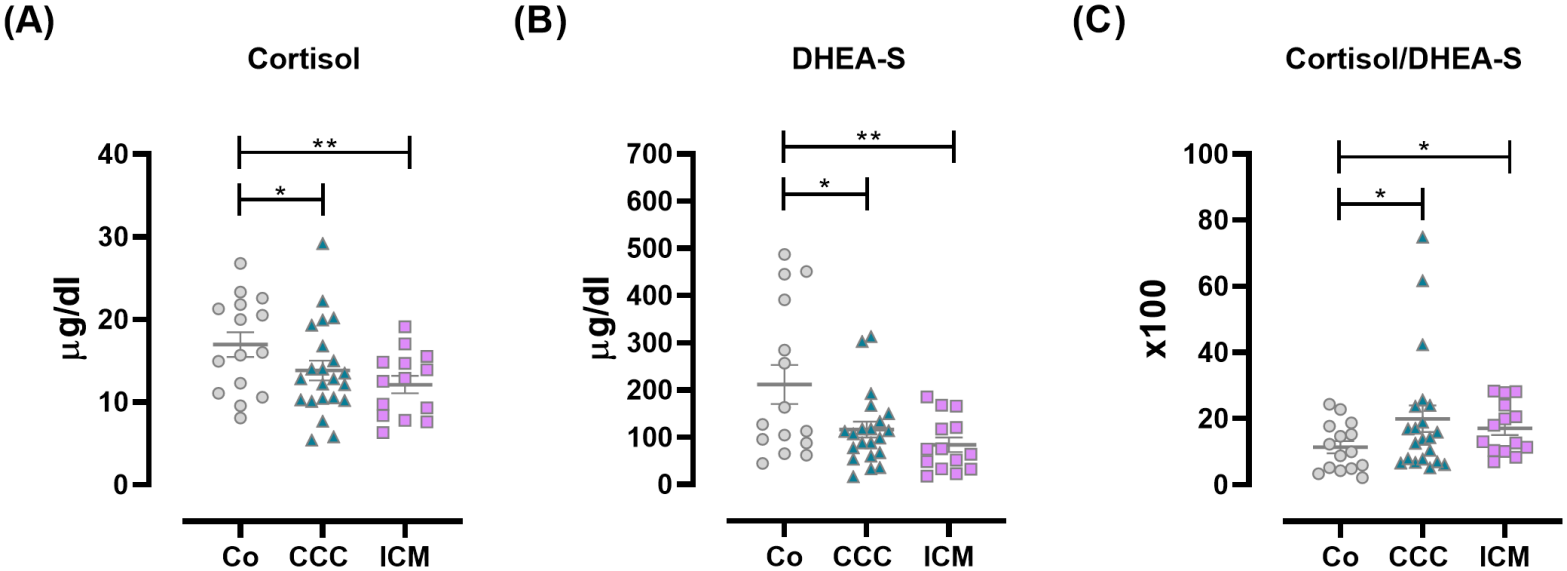
Plasma levels of HPA-axis-related factors in Co, CCC, and ICM patients. **(A)** Cortisol, **(B)** DHEA-S, and **(C)** Cortisol/DHEA-S ratio. Lines represent means ± SEM. *p<0.05; **p<0.01.

Our earlier study (3) revealed that CCC patients have dysregulated HPA-axis activation, indicated by low-normal levels of cortisol and DHEA-S, resulting in an increased cortisol/DHEA-S ratio. Here, to establish whether the increased cortisol/DHEA-S ratio is related to Chagas disease or merely the presence of cardiomyopathy, we compared this ratio with that in ICM counterparts. As previously reported, both cortisol and DHEA-S levels were decreased in CCC patients, similar to the ICM group (**Figures 1A, B**). An increased cortisol/DHEA-S ratio (**Figure 1C**) was observed in CCC patients, and to a lesser extent, in ICM subjects.

### The pro-inflammatory pattern in PBMCs from CCC patients is associated with changes in 11β-HSD1 and TTP expression but not GR-α

**Figure 2:**
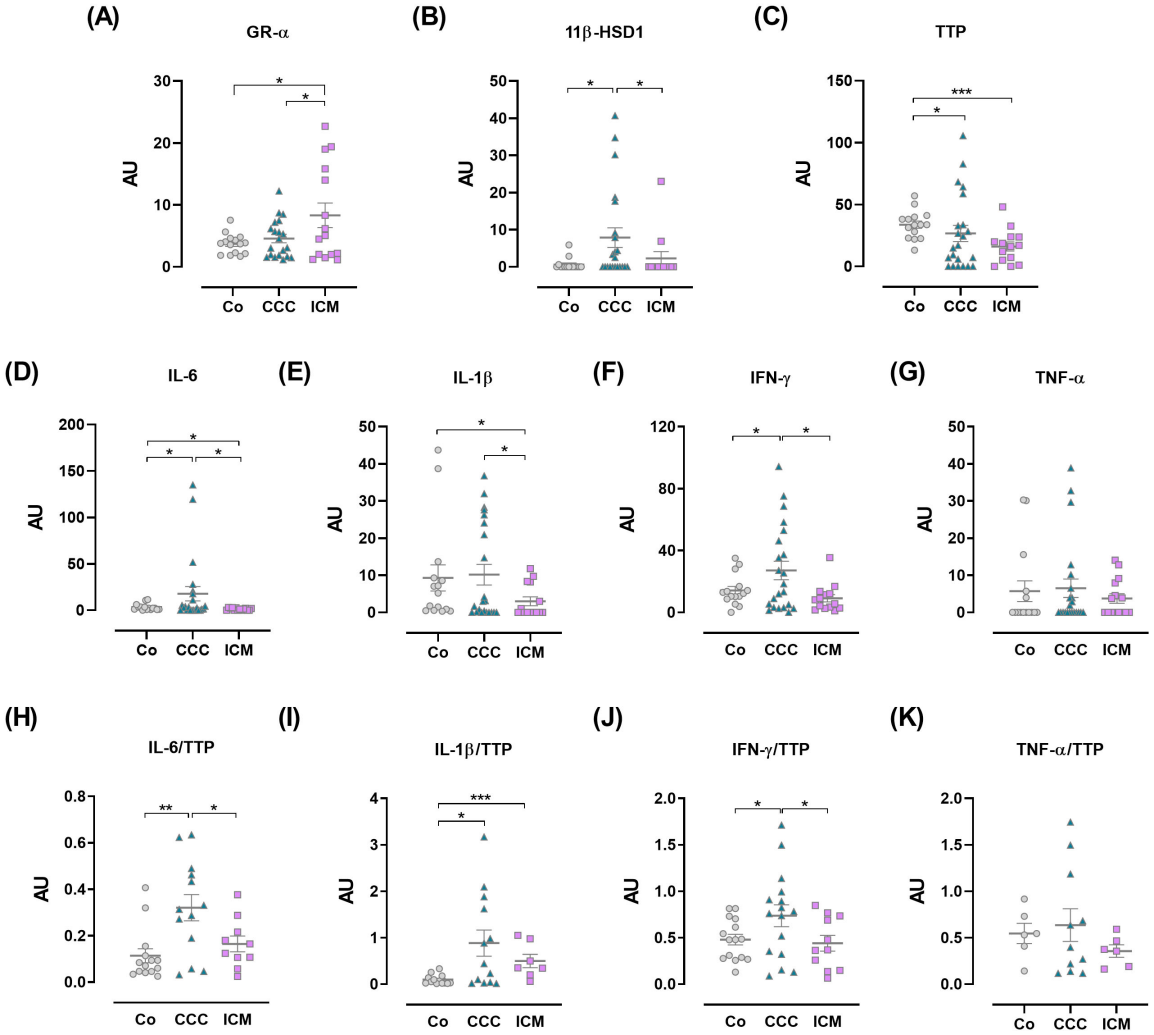
Expression of immuno-endocrine-related genes in peripheral blood mononuclear cells. **(A)** Glucocorticoid receptor-α (GR-α). **(B)** 11β-hydroxysteroid dehydrogenase-1 (11β-HSD1). **(C)** Tristetraprolin (TTP). **(D)** IL-6. **(E)** IL-1β. **(F)** IFN-γ. **(G)** TNF-α. **(H)** IL-6/TTP ratio. **(I)** IL-1β/TTP ratio. **(J)** IFN-γ/TTP ratio. **(K)** TNF-α/TTP ratio. The results are expressed as arbitrary units (AU). Lines represent means ± SEM. *p<0.05; **p<0.01; ***p<0.001.

Within the systemic bidirectional communication between pro- and anti-inflammatory factors, PBMCs were analyzed for the expression of GR-α and GR-β, 11β-HSD1, GR-regulated genes like TTP, and pro-inflammatory cytokines. Since the GR-α/GR-β ratio can influence GC response, the expression of GR isoforms in PBMCs likely indicates GC sensitivity. As shown in **Figure 2A**, ICM individuals exhibited increased GR-α expression compared to other groups. Although GR-β expression was detectable in positive control samples, it was undetectable in PBMCs from patients or controls, implying that the amplification was specific. Still, PBMCs either did not express or expressed undetectable levels of this isoform (**Supplementary Table S5**).

GCs actions go beyond their circulating level, as their bioavailability can be controlled by the local intracellular enzyme 11β-HSD1. To further assess the status of immune cells in terms of tissue GC bioavailability we evaluated the expression of the 11β-HSD1. PBMC from patients with CCC showed significantly increased 11β-HSD1 expression compared to PBMCs from Co and those with ICM (**Figure 2B**).

Given that TTP is upregulated by GR-α activation and plays a role in the counter-regulatory response, its expression in PBMCs was also evaluated. TTP mRNA amounts appeared lower in the CCC and ICM groups, with the former group showing greater variations (**Figure 2C**).

Moreover, PBMCs from CCC patients exhibited increased IL-6 and IFN-γ expression compared to Co and ICM patients, with a tendency towards higher IL-1β expression (**Figures 2D–F**). In contrast, ICM PBMCs showed decreased expression of these cytokines compared to the Co group (**Figures 2D, E**). TNF-α levels did not show significant variations (**Figure 2G**). Considering that TTP binds to pro-inflammatory cytokine mRNAs promoting their degradation, we analyzed the expression ratios between cytokines and TTP, assuming that an increased cytokine/TTP ratio may indicate inadequate production of TTP in response to increased inflammatory cytokine levels (29). Data show elevated ratios of IL-6/TTP and IFN-γ/TTP in CCC patients compared to Co and ICM groups (**Figures 2H, J**), while the IL-1β/TTP ratio was increased in both CCC and ICM PBMCs compared to Co (**Figure 2I**). PBMCs from CCC individuals also exhibited a trend towards an elevated TNF-α/TTP ratio (**Figure 2K**).

### Correlations between hormones and transcripts

Correlation studies were performed to examine the link between endocrine and immune parameters in disease pathology. Results are outlined in **Supplementary Table S6** and **Supplementary Figure S1**. Notably, certain physiological correlations seen in Co were absent in CCC and ICM patients. In the CCC group, 11β-HSD1 enzyme expression showed a positive correlation with IL-1β and cytokine/TTP ratio.

### GR expression is increased in CCC cardiomyocytes and associated with hypertrophy

**Figure 3:**
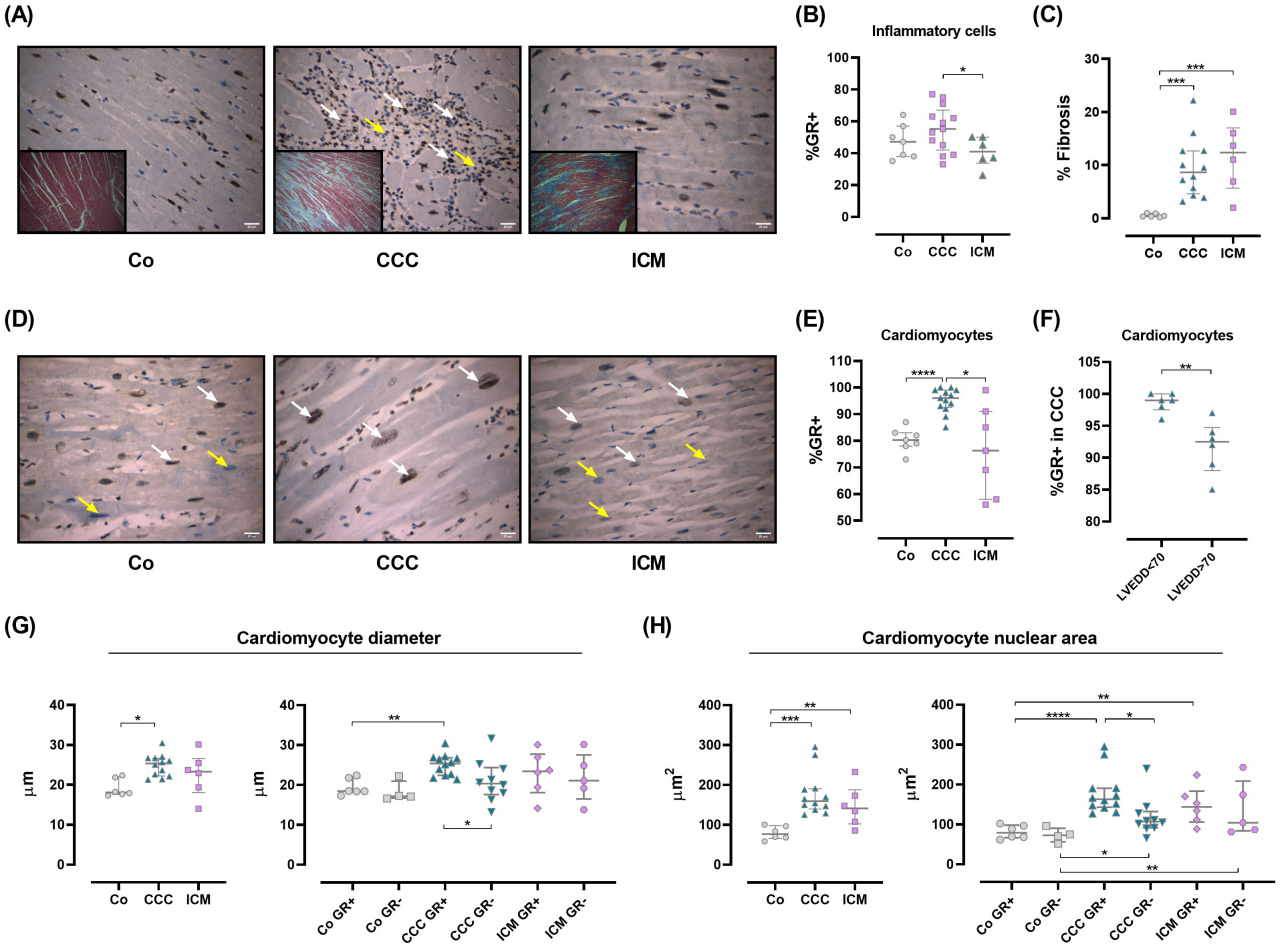
Glucocorticoid receptor expression in human cardiac tissue samples. **(A)** Representative images of hematoxylin/eosin staining from the interventricular septum in Co, CCC, and ICM individuals (O.M 400X). A diffuse inflammatory infiltrate is observed in CCC patients (middle panel, white arrows). Inserts show fibrosis for each group. **(B)** Percentage of GR+ inflammatory cells in 10 HPF (O.M 400X). **(C)** Fibrosis as a percentage of the total area. **(D)** GR localization and immunoreactivity. GR+ cells (brown) are mainly nuclear (white arrows); yellow arrows indicate GR-negative cells (light blue). **(E)** GR+ expression in cardiomyocytes, quantified in 10 HPF (O.M 400X). **(F)** GR+ expression in CCC patients, categorized by LVEDD. **(G)** Cardiomyocyte diameters (right panel) and comparison by GR expression (left panel). **(H)** Cardiomyocyte nuclear areas (right panel) and comparison by GR expression (left panel). Data are means ± SEM. *p<0.05; **p<0.01; ***p<0.001; ****p<0.0001. HPF, high power field; O.M., original magnification.

CCC is a chronic inflammatory and fibrosing myocarditis whose pathophysiology remains controversial, although parasite persistence along with adverse immune reactions lead to tissue damage (2). Since GCs may influence the trafficking of infiltrating immune cells, the cardiomyocyte responses, and given that their circulating levels are slightly diminished, the GR expression in cardiac tissue samples from CCC, ICM, and Co subjects was evaluated as a mode to mirror GC sensitivity. Hence, we conducted a histological analysis and immunohistochemistry to detect GR in heart tissues from Co, ICM, and CCC subjects. As known, the CCC myocardium shows a notable inflammatory component with increased inflammatory infiltrates (**Figure 3A** and **Supplementary Table S4**) (30). Even inflammatory cells showed an increased expression of GR (**Figure 3B**).

Fibrosis is evident in CCC and ICM hearts (**Figures 3A, C**). When analyzing GR expression in cardiomyocytes, it was clear that the CCC group expressed higher levels of GR than the remaining groups (**Figures 3D, E**). Particularly, within this group the major GR expression was found in cardiomyocytes from patients with left ventricular end-diastolic diameter (LVEDD) below 70 mm (**Figure 3F**). The relationship between GR expression and cardiomyocyte hypertrophy was examined by measuring cardiomyocyte diameters and nuclear areas. In CCC, cardiomyocyte diameters increased, particularly in GR-expressing cells (**Figure 3G**). Similarly, higher nuclear areas were seen in CCC cardiomyocytes expressing GR (**Figure 3H**). Conversely, in the ICM group, nuclear areas increased without a clear link to GR expression (**Figure 3H**).

### Correlations between immunohistochemistry and cardiac morphometric parameters

As shown in **Supplementary Table S7** and **Supplementary Figure S2**, cardiac weight shows a positive correlation with myocardial fibrosis (overall, rho=0.622; p=0.001), total nuclear area (overall, rho=0.467; p=0.021), and nuclear area of GR+ cardiomyocytes (overall, rho=0.453; p =0.026). Specifically, in the CCC group, cardiac weight negatively correlates with GR expression in inflammatory infiltrates (rho=-0.580; p=0.048). GR+ in the inflammatory infiltrate positive correlates with GR+ in cardiomyocytes (overall, rho= 0.596; p=0.002 and CCC, rho=0.550; p=0.05) and nuclear area (overall, rho=0.482; *p*=0.017). Additionally, the intensity of the inflammatory infiltrate positively correlates with GR+ in cardiomyocytes (overall, rho=0.685; p<0.01) and the nuclear area (overall, rho=0.465; p=0.022).

An interesting finding, as seen in **Supplementary Table S7** and **Supplementary Figure S2B** was that the LVEDD in patients with CCC was directly correlated with cardiac weight (rho=0.604; p=0.038) and inversely with the expression of GR+ in cardiomyocytes (rho=-0.746; p=0.005) and the inflammatory infiltrate (rho=-0.853; p<0.001).

## Discussion

The role of GR activation in different pathologies is still controversial. In human pathological circumstances such as adrenal tumors or Cushing’s syndrome, hypercortisolemia has been associated with adverse cardiovascular outcomes, including hypertrophy and myocardial remodeling (31–33). However, it is important to remark that these situations are studied in the absence of infectious processes like Chagas disease, where the onset of heart damage is different.

Although systemic GC levels were slightly decreased in CCC patients, GR expression was augmented in their cardiomyocytes, potentially as an attempt to respond more efficiently to GCs. This increased GR expression was associated with cell hypertrophy, while cardiac weight correlated positively with the nuclear area of GR+ cardiomyocytes.

The increase in GR expression in hypertrophic cardiomyocytes may be a consequence of the augmented inflammatory infiltration and fibrosis, as this phenomenon is not observed in ICM hearts, which lack immune cell infiltrations. In this sense, a positive correlation between the intensity of inflammatory infiltrate and cardiomyocyte GR expression was found. In the CCC group, inflammatory infiltrating cells also expressed higher levels of GR, and GR expression in these cells positively correlated with GR expression in cardiomyocytes and their nuclear area. Thus, in the context of CCC, GR expression seems to be a common feature across all cells, enhancing GC sensitivity to manage chronic inflammation, but with the potential detrimental outcome of hypertrophy (13). Given that GR expression was predominantly found in the nucleus of cardiomyocytes, this may indicate that the GR/GC complex has migrated to the nucleus to activate downstream GR target genes that promote cardiomyocyte hypertrophy. On the other hand, we also found that CCC patients with LVEDD below 70 presented a higher percentage of GR expression, which leads us to hypothesize that the expression of GR decreases as the condition progresses from adaptive hypertrophy to terminal dilated cardiomyopathy. These findings align with the majority of experimental studies, which demonstrate that in transgenic KO mouse models for GR expression, GR deletion results in cardiac hypertrophy, dilation, and systolic dysfunction associated with heart failure (17).

Another issue worth considering is the difficulty in distinguishing between GR-α and GR-β expression in human myocardial tissue due to the current lack of reliable antibodies. Furthermore, technical limitations prevent us from measuring their transcripts in fixed cardiac tissues, which represents a limitation of our approach. However, if the increase in GR expression in hypertrophic cells was due to an increase in the GR-β isoform, cardiac hypertrophy might be mediated by GR deficiency. In this regard, genome-wide microarray analysis of hearts from GR knock-out mice revealed several “cardiovascular disease”-related genes differentially expressed. Notably, there was significantly reduced expression of genes involved in inhibiting myocardial hypertrophy (Klf15), promoting cell survival (prostaglandin D2 synthase, Ptgds), and restraining inflammation (lipocalin 2, Lcn2; TTP) (17).

Regarding GR activation in PBMCs from CCC patients, the downstream regulation of GR appears to be impaired. TTP expression is decreased, while pro-inflammatory cytokines tend to increase, leading to an augmented pro-inflammatory cytokine/TTP ratio that reflects the failure to compensate for systemic inflammation in these patients. Correlation analysis also reflects a counteracting response attempting to compensate for the pro-inflammatory milieu. This is in line with our earlier studies, reporting increased expression of anti-inflammatory receptors such as adiponectin receptor and PPAR-γ in PBMCs from CCC patients (3).

It is known that 11β-HSD1 transcripts, protein, and enzyme activities are actively expressed in murine T and B lymphocytes. Additionally, activation of CD4+ T-cells increases 11β-HSD1 activity, and GCs generated by this enzyme can engage and activate the GR (34). Thus, the presence of 11β-HSD1 in PBMCs and inflamed tissues likely constitutes an additional mechanism to facilitate GC influences over lymphocyte activities, regardless of its plasma concentration.

Moreover, it may endow lymphocytes with an intracrine regulation capable of influencing processes such as lymphocyte development or effector function. In PBMCs from CCC patients, the expression of 11β-HSD1 is increased, possibly as an attempt to augment cellular GC availability and counteract the inflammatory environment in the context of low-normal GC plasma levels. This increased expression of 11β-HSD1 was also found in pleural effusion mononuclear cells from patients with tuberculosis, suggesting a local effort to optimize the immunomodulatory properties of reduced cortisol concentrations in an inflammatory setting (35).

Additionally, inflammation can upregulate the expression and activity of 11β-HSD1 in rat lymph nodes and spleen in a model of trinitrobenzene sulfonic acid-induced colitis. In these studies, increased expression of inflammation markers coincided with heightened 11β-HSD1 expression, especially in intraepithelial and lamina propria lymphocytes. This suggests regional effector cells display a higher activation of the GC regenerating system (36). However, in human CCC cardiac samples, 11β-HSD1 expression was undetectable. In patients with CCC and other chronic inflammatory conditions, dysregulation of the HPA axis leads to reduced cortisol and DHEA-S secretion (3, 37–39). Studies suggest that this deficiency can stem from elevated TNF-α levels, triggering widespread upregulation of 11β-HSD1 expression, even in the hypothalamus. This amplifies negative feedback by GCs on the HPA axis (40). The increased 11β-HSD1 expression in PBMCs of CCC patients may mirror similar processes in other tissues, warranting further investigation.

**Figure 4:**
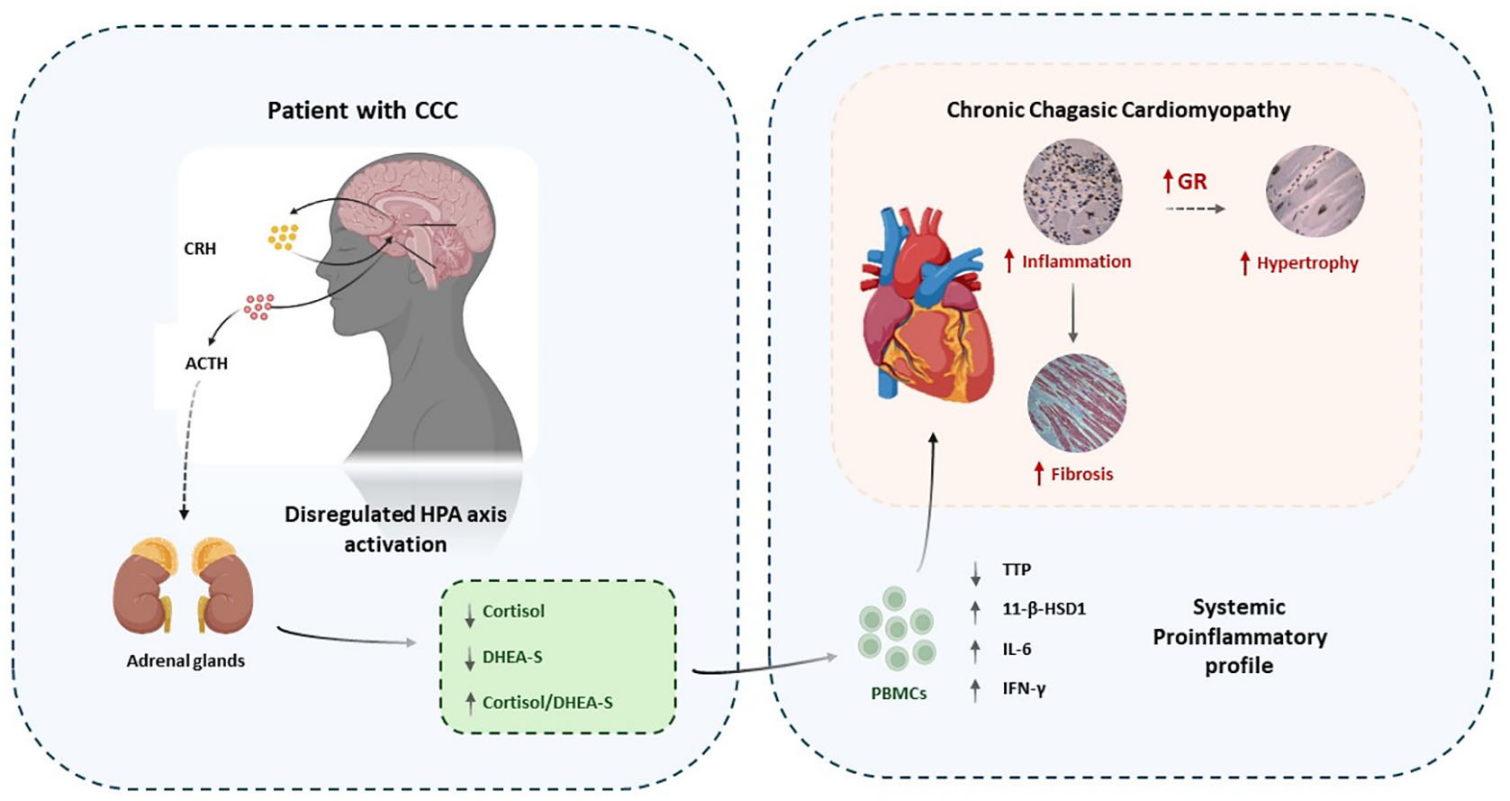
Conceptual graph showing the association between endocrine dysregulation, inflammation, GR expression and CCC pathology. Immune-endocrine imbalances and parasite persistence may favor the establishment of a chronic proinflammatory state in parallel with cardiac inflammation. Cardiomyocytes might upregulate GR expression to counter inflammation which could favor the development of hypertrophy. At the same time fibrosis would develop in response to tissue damage due to inflammation. .

The dysregulation of the crosstalk between the endocrine and immune systems in CCC patients appears to contribute to the maintenance of a pro-inflammatory profile, as evidenced by the imbalanced expression of genes regulated by the GR in PBMCs. This dysregulation likely plays a significant role in the infectious and inflammatory nature of CCC pathology, potentially linked to variations in GR expression in cardiac tissue samples. Considering that there are no other studies, to the best of our knowledge, evaluating the expression of GR in human cardiac tissue samples, these findings offer a stimulating background for future research aimed at exploring the impact of GR expression and function on CCC pathophysiology, particularly concerning cardiomyocyte hypertrophy (**Figure 4**).

## Data Availability

The raw data supporting the conclusions of this article will be made available by the authors, without undue reservation.
