# Supplementary material for: Role of glucocorticoid receptor expression in Chronic Chagas Cardiomyopathy: implications for inflammation and cardiac hypertrophy
Source: Front Endocrinol (Lausanne). 2025 Jan 30;16:1486772. doi: 10.3389/fendo.2025.1486772 (PMC11821487; doi:10.3389/fendo.2025.1486772)
Supplement: Supplementary file 1 [file DataSheet1.docx]

**SUPPLEMENTARY MATERIALS**

**Table-S1. Characteristics of patients with chronic Chagas disease, Ischemic Cardiomyopathy, and Control individuals included in PBMCs studies.**

| **Variable** | **Controls**  **(n=15)** | **CCC**  **(n=22)** | **ICM**  **(n=14)** |
| --- | --- | --- | --- |
| Age (years ± SD) | 43.2 ± 16.17 | 49.9 ± 14.10 | 53.0 ± 7.28 |
| Range, min-max | 17 – 64 | 18 – 66 | 33 - 64 |
| Sex (males, n %) | 5 (33%) | 4 (18%) | 8 (57%) |
| Body Mass Index (kg/m2 ± SD) | 24.1 ± 2.94 | 30.6 ± 5.18 | 28.2 ± 4.46 |
| Left ventricular ejection fraction (% ± SD) | 63.0 ± 5.10 | 50.9 ± 9.60 | 25.8 ± 12.67 |
| Left ventricular end-diastolic diameter, (mm ± SD) | 52.2 ± 2.13 | 59.60 ± 6.84 | 65.20 ± 16.42 |
| Systolic blood pressure (mm Hg ± SD) | 124.1 ± 5.80 | 125.8 ± 17.69 | 120.6 ± 19.46 |
| Diastolic blood pressure (mm Hg, ± SD) | 73.2 ± 1.23 | 76.25 ± 9.32 | 78.17 ± 12.48 |

Continuous data are shown as means ± standard deviation. Categorical data are shown as numbers and (%). ICM = Ischemic Cardiomyopathy; CCC= Chronic Chagasic Cardiomyopathy.

**Table-S2. Demographic, clinical, and pathologic characteristics of chronic Chagas disease or Ischemic Cardiomyopathy patients, and Control individuals including in histology and immunohistochemistry of cardiac tissue assays.**

| **Variable** | **Controls**  **(n=6)** | **ICM**  **(n=6)** | **CCC**  **(n=12)** | **p-value** |
| --- | --- | --- | --- | --- |
| Age (years ± SD) | 43.5 ± 15.2 | 57.8 ± 8.0 | 53.3 ± 7.7 | 0.059 |
| Gender (males, n %) | 2 (33%) | 3 (50%) | 7 (58%) | 0.335 |
| Body mass index (kg/m2 ± SD) | 26.6 ± 4.0 | 24.2 ± 4.9 | 26.0 ± 4.3 | 0.615 |
| Left ventricular ejection fraction (% ± SD) | NA | 29.5 ± 15.6 | 23.3 ± 10.2 | 0.644 |
| Left ventricular end-diastolic diameter, (mm ± SD) | NA | 67.17 ± 9.9 | 69.25 ± 4.6 | 0.644 |
| Heart weight (g ± SD) | 240.0 ± 27.6 | 496.7 ± 111.3 | 435.4 ± 74.1 | <0.001 |
| Thickness of ventricular wall (mm ± SD) |  | | | |
| *- Right ventricular* | 4.00 ± 0.6 | 3.17 ± 0.4 | 4.17 ± 1.6 | 0.264 |
| *- Interventricular Septum* | 13.17 ± 2.3 | 11.17 ± 5.2 | 11.42 ± 2.8 | 0.533 |
| *- Anterior LV* | 11.33 ± 1.2 | 7.5 ± 3.3 | 8.6 ± 2.7 | 0.050 |
| *- Lateral LV* | 11.67 ± 0.8 | 10.50 ± 3.1 | 7.92 ± 3.0 | 0.023 |
| *- Posterior LV* | 11.33 ± 2.3 | 10.00 ± 4.4 | 8.58 ± 2.9 | 0.241 |
| Interstitial fibrosis (% ± SD) | 0.55 ± 0.34 | 11.62 ± 6.48 | 9.58 ± 5.61 | 0.002 |
| Interstitial infiltrate cells (n, median IQR) | 37 (36 – 46) | 35 (18 – 39) | 829 (602 – 966) | <0.001 |
| Continuous data are shown as means ± standard deviation (SD). Categorical data are shown as numbers (%). ICM = Ischemic Cardiomyopathy; CCC = Chronic Chagasic Cardiomyopathy; LV = left ventricle; IQR = interquartile range; NA = not available. | | | | |

**Table-S3. Primer sequences used for qPCR analysis in PBMC samples.**

| **Gene** | **Primer sequence** | |
| --- | --- | --- |
|  |  |  |
| Cyclophilin A | F: 5′-GGTCCTGGCATCTTGTCCAT-3′ | R: 5′-TTGCTGGTCTTGCCATTCCT-3′ |
| RG-α | F: 5’-GAAGGAAACTCCAGCCAGAAC-3’ | R: 5’-GATGATTTCAGCTAACATCTCG-3’ |
| RG-β | F: 5’-GAAGGAAACTCCAGCCAGAAC-3’ | R: 5’-TGAGCGCCAAGATTGTTGG-3’ |
| 11β-HSD1 | F: 5’-ATGATATTCACCATGTGCGCA-3’ | R: 5’- ATAGGCAGCAACCATTGGATAAG-3’ |
| IL-6 | F: 5’-TACGGCGCTGTCATCGAT-3’ | R: 5’-TAGAGTCGCCACCCTGATGT-3’ |
| IL-1β | F: 5’-TCTGTACCTGTCCTGCGTGTTG-3’ | R: 5’-GGGGAACTGGGCAGACTCAA -3 |
| TTP | F: 5’-ATGGCCAACCGTTACACC-3’ | R: 5’-TCCATGGTCGGATGGCAC-3’ |
| TNF-α | F: 5′-AGCCCATGTTGTAGCAAACCC-3′ | R: 5′-GTTATCTCTCAGCTCCACGCC-3′ |
| IFN-γ | F: 5’-AACGAGATGACTTCGAAAAGCTG-3’ | R: 5’-TCTTCGACCTCGAAACAGCA-3’ |

**Table-S4. Staining Procedure of Glucocorticoid Receptor**

| **Procedure Parameter** | **Selection** |
| --- | --- |
| Deparaffinization | Selected |
| Cell Conditioning | CC1 Cell Conditioning 40 min (Roche Nr.950-124) |
| Pre-primary antibody peroxidase Inhibitor | Selected |
| Antibody (Primary) | Anti-GR (Clon 41-BD#611226) 40 min |
| OptiView HQ Linker | 8 min(Roche Nr.760-700) |
| OptiView HRP Multimer | 8 min |
| OptiView Amplification | Not selected |
| Counterstain | Hematoxylin II, 12 min |
| Post Counterstain | Bluing Reagent, 4 min |

**Table-S5. Expression of GR-β in PBMC samples.**

| **Group** | **ID Sample** | **CT** |
| --- | --- | --- |
| Control | 1-14 | Not detectable |
| CCC | 15-37 | Not detectable |
| ICM | 38-51 | Not detectable |
| Curve | Positive control 1 | 32 |
| Curve | Positive control 2 | 34 |
| Curve | Positive control 3 | 36 |
| Curve | Positive control 4 | 38 |

**Tabla-S6.** ***Correlation analysis between circulating levels of hormones and RNAm transcripts***

|  | **Overall** | | **Controls** | | **CCC** | | **ICM** | |
| --- | --- | --- | --- | --- | --- | --- | --- | --- |
| **Correlations** | **rho** | ***p-value*** | **rho** | ***p-value*** | **rho** | ***p-value*** | **rho** | ***p-value*** |
| **CT/DHEA-S vs TTP** | -0.225 | 0.1127 | -0.625 | 0.015 | NS | NS | NS | NS |
| **CT/DHEA-S vs IFN-ϒ/TTP** | -0.101 | 0.4819 | NS | NS | -0.5647 | 0.024 | NS | NS |
| **CT/DHEA-S vs IL-1β** | -0.345 | 0.0132 | NS | NS | -0.4252 | 0.048 | NS | NS |
| **DHEA-S vs IL-1β** | 0.324 | 0.0202 | 0.532 | 0.044 | NS | NS | NS | NS |
| **DHEA-S vs TNF-α** | 0.119 | 0.4052 | 0.640 | 0.012 | NS | NS | NS | NS |
| **DHEA-S vs IL-1β/TTP** | 0.313 | 0.0256 | 0.525 | 0.047 | 0.6519 | 0.007 | NS | NS |
| **DHEA-S vs TNF-α/TTP** | 0.083 | 0.5636 | 0.636 | 0.013 | NS | NS | NS | NS |
| **RG-α vs IL-1β** | 0.061 | 0.6695 | 0.546 | 0.038 | NS | NS | NS | NS |
| **RG-α vs CT** | -0.327 | 0.0191 | -0.593 | 0.022 | NS | NS | NS | NS |
| **RG-α vs TTP** | 0.410 | 0.0028 | NS | NS | 0.4859 | 0.021 | NS | NS |
| **RG-α vs IFN-ϒ** | 0.581 | <0.0001 | NS | NS | 0.5313 | 0.010 | 0.833 | 0.000 |
| **IFN-ϒ vs CT** | -0.196 | 0.1687 | -0.779 | 0.001 | NS | NS | NS | NS |
| **IFN-ϒ vs TTP** | 0.583 | <0.0001 | NS | NS | 0,6621 | 0.000 | 0.568 | 0.037 |
| **TTP vs IL-1β** | 0.426 | 0.0018 | 0.632 | 0.013 | NS | NS | NS | NS |
| **TTP vs IL-6** | 0.568 | <0.0001 | NS | NS | 0,6650 | 0.001 | 0.524 | 0.057 |
| **TTP vs TNF-α** | 0.346 | 0.0128 | 0.532 | 0.044 | 0.4852 | 0.0221 | 0.524 | 0.057 |
| **11β-HSD1 vs IL-1β** | 0.432 | 0.0015 | NS | NS | 0.5316 | 0.011 | 0.525 | 0.036 |
| **11β-HSD1 vs IL-6** | 0.557 | <0.0001 | NS | NS | 0,7388 | <0.0001 | NS | NS |
| **11β-HSD1 vs TNF-α** | 0.715 | <0.0001 | NS | NS | 0.8948 | <0.0001 | 0.491 | 0.045 |
| **11β-HSD1 vs IL-6/TTP** | 0.613 | <0.0001 | NS | NS | 0.7969 | <0.0001 | NS | NS |
| **11β-HSD1 vs TNF-α/TTP** | 0.743 | <0.0001 | NS | NS | 0.9343 | <0.0001 | 0.532 | 0.034 |
| **11β-HSD1 vs IL-1β/TTP** | 0.521 | <0.0001 | NS | NS | 0.6705 | 0.0006 | NS | NS |
| **11β-HSD1 vs IFN-ϒ/TTP** | 0.409 | 0.0029 | NS | NS | 0.6393 | 0.0014 | NS | NS |

rho: Spearman’s coefficient of correlation. NS: not significant. CT: cortisol

**Tabla-S7. *Correlation analysis between immunochemistry and morphological parameters in heart tissue.***

|  | **Overall** | | **Controls** | | **CCC** | | **ICM** | |
| --- | --- | --- | --- | --- | --- | --- | --- | --- |
| ***Correlation*** | **rho** | ***p-value*** | **rho** | ***p-value*** | **rho** | ***p-value*** | **rho** | ***p-value*** |
| Cardiac weight vs myocardial fibrosis | 0.622 | 0.001 | NS | NS | NS | NS | NS | NS |
| Cardiac weight vs nuclear area | 0.467 | 0.021 | NS | NS | NS | NS | NS | NS |
| Cardiac weight vs nuclear area of GR+ cardiomyocytes | 0.453 | 0.026 | NS | NS | NS | NS | NS | NS |
| Cardiac weight vs expression of GR in the inflammatory infiltrate | NS | NS | NS | NS | -0.580 | 0.048 | NS | NS |
| GR expression in the inflammatory infiltrate vs myocardial fibrosis | NS | NS | NS | NS | NS | NS | NS | NS |
| Intensity of the inflammatory infiltrate vs myocardial fibrosis | NS | NS | NS | NS | NS | NS | NS | NS |
| Expression of GR in the inflammatory infiltrate vs nuclear area of the cardiomyocytes | 0.482 | 0.017 | NS | NS | NS | NS | NS | NS |
| Intensity of the inflammatory infiltrate vs nuclear area of the cardiomyocytes | 0.465 | 0.022 | -0.816 | 0.04 | NS | NS | NS | NS |
| Expression of GR in the inflammatory infiltrate vs expression of GR in cardiomyocytes | 0.596 | 0.002 | NS | NS | 0.550 | 0.05 | NS | NS |
| Intensity of the inflammatory infiltrate vs expression of GR in cardiomyocytes | 0.685 | <0.001 | NS | NS | NS | NS | NS | NS |
| Expression of GR in cardiomyocytes vs cardiomyocytes nuclear area | 0.482 | 0.017 | NS | NS | NS | NS | NS | NS |
| LVEDD vs cardiac weight | 0.562 | 0.015 | NA | NA | 0.604 | 0.038 | NS | NS |
| LVEDD vs expression of GR in cardiomyocytes | NS | NS | NA | NA | -0.746 | 0.005 | NS | NS |
| LVEDD vs expression of GR in the inflammatory infiltrate | NS | NS | NA | NA | -0.853 | <0.001 | NS | NS |

rho: Spearman’s coefficient of correlation. NS: not significant. NA: not available. GR: glucocorticoid receptor.


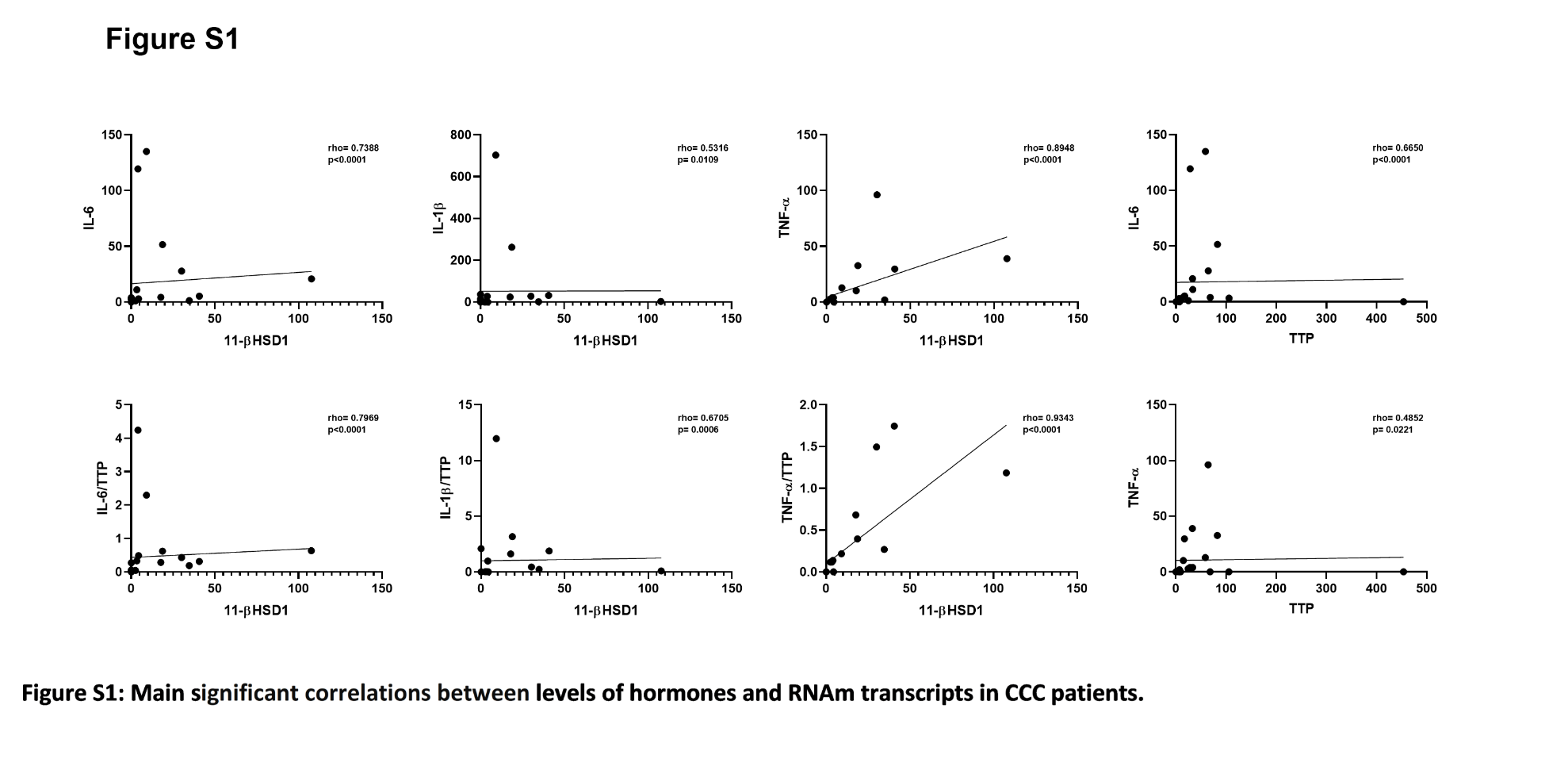


**Figure S1: Significant correlations between mRNA transcripts in CCC patients.**

**
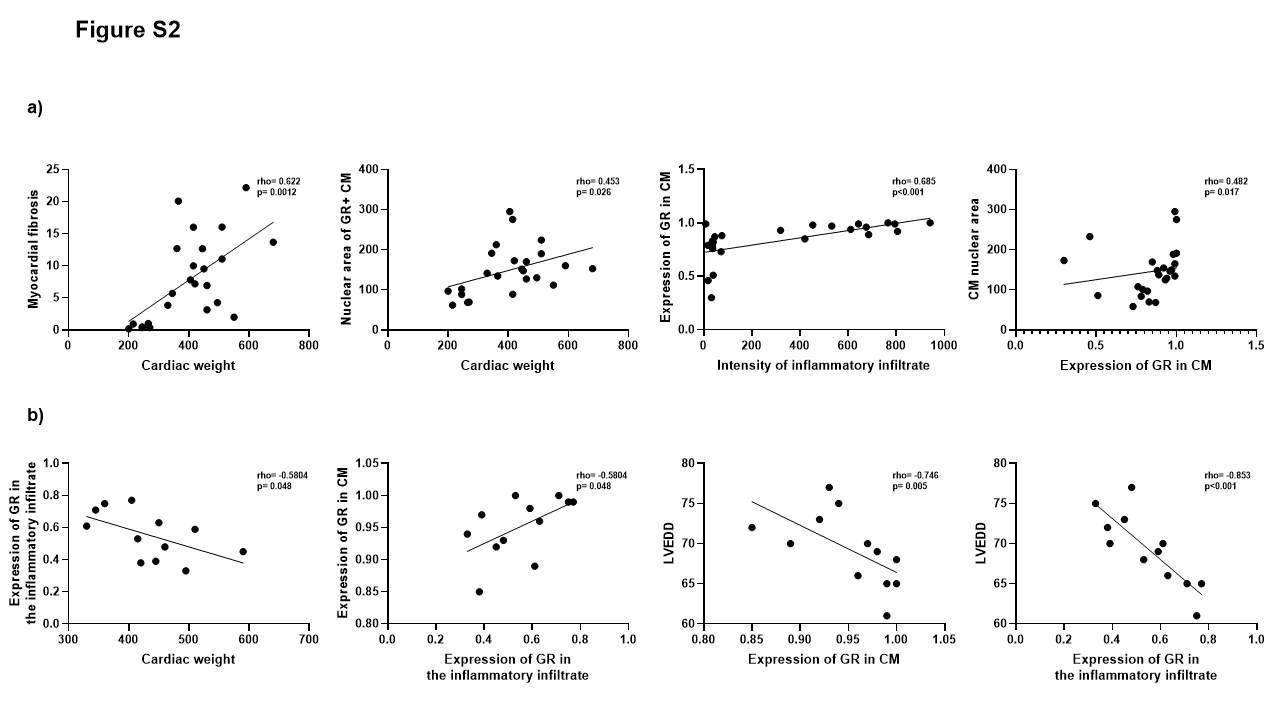
**

**Figure S2:** **Significant correlations between immunohistochemistry and cardiac morphometric parameters.** a) Overall population. b) CCC patients
